# (*R*)-10-Hydroxystearic Acid: Crystals vs. Organogel

**DOI:** 10.3390/ijms21218124

**Published:** 2020-10-30

**Authors:** Fioretta Asaro, Carla Boga, Rita De Zorzi, Silvano Geremia, Lara Gigli, Patrizia Nitti, Sabrina Semeraro

**Affiliations:** 1 Department of Chemical and Pharmaceutical Sciences, University of Trieste, via L. Giorgieri 1, 34127 Trieste, Italy; sgeremia@units.it (S.G.); pnitti@units.it (P.N.); ssemeraro@units.it (S.S.); 2 Department of Industrial Chemistry “Toso Montanari”, University of Bologna, Viale del Risorgimento 4, 40136 Bologna, Italy; carla.boga@unibo.it; 3 Elettra–Sincrotrone Trieste, S.S. 14 Km 163.5 in Area Science Park, Basovizza, 34149 Trieste, Italy; lara.gigli@elettra.eu

**Keywords:** crystal structure, IR spectroscopy, organogel, (*R*)-hydroxystearic acid, polymorphism, X-ray diffraction

## Abstract

The chiral (*R*)-10-hydroxystearic acid ((*R*)-10-HSA) is a positional homologue of both (*R*)-12-HSA and (*R*)-9-HSA with the OH group in an intermediate position. While (*R*)-12-HSA is one of the best-known low-molecular-weight organogelators, (*R*)-9-HSA is not, but it forms crystals in several solvents. With the aim to gain information on the structural role of hydrogen-bonding interactions of the carbinol OH groups, we investigated the behavior of (*R*)-10-HSA in various solvents. This isomer displays an intermediate behavior between (*R*)-9 and (*R*)-12-HSA, producing a stable gel exclusively in paraffin oil, while it crystallizes in other organic solvents. Here, we report the X-ray structure of a single crystal of (*R*)-10-HSA as well as some structural information on its polymorphism, obtained through X-ray Powder Diffraction (XRPD) and Infrared Spectroscopy (IR). This case study provides new elements to elucidate the structural determinants of the microscopic architectures that lead to the formation of organogels of stearic acid derivatives.

## 1. Introduction

**Figure 1:**
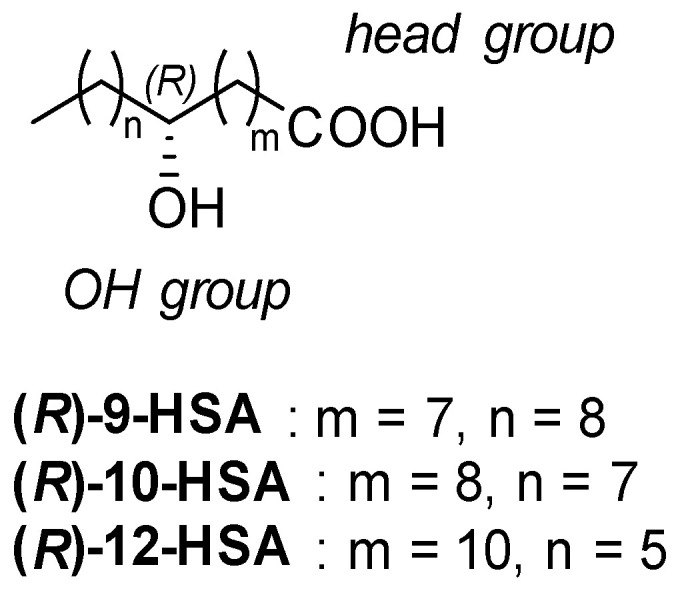
Structures of (*R*)-9-hydroxystearic acid (HSA), (*R*)-10-has, and (*R*)-12-HSA, hydroxystearic acids (HSA) with the OH group at positions 9, 10, and 12, respectively.

Hydroxy fatty acids are very appealing compounds because they are structurally simple and have a large number of constitutional and optical isomers, since the hydroxyl group may be located at any position along the alkyl chain (Figure 1).

These molecules possess two sites capable of forming hydrogen-bonding interactions, namely the carboxylic head group and the alcoholic OH group. In addition, they can be chiral when the OH group is located on an internal carbon of the alkyl chain. Thus, these compounds give rise to intriguing chiral supramolecular architectures, both in water and in organic solvents [1,2,3]. One of the first reported examples are the helical fibers observed for the lithium salt of (*R*)-12-hydroxystearic acid ((*R*)-12-HSA)) in mineral oil [4].

Nowadays, (*R*)-12-HSA is the hydroxy fatty acid of the most widespread use. It is employed as a gelator and thickener of oils and organic solvents in a wide range of products, including lubricants, paints, cosmetics, and food. Another important aspect is that its industrial production consists in the hydrogenation of renewable sources, such as ricinoleic acid, the most abundant fatty acid of castor oil [5].

(*R*)-12-HSA owes its activity as a low-molecular-weight organogelator (LMWO) to its ability to form self-assembled networks able to entrap the liquid phase of many organic solvents. In this process, the chirality of the tail plays a paramount role, as the gelation properties at low concentration are exclusively present in the enantiomerically pure form, while the racemic mixture is active only at much higher concentrations [6].

Organogels of (*R*)-12-HSA are obtained by cooling a hot solution prepared with a solvent where the hydroxy fatty acid is insoluble at a lower temperature. Solvent, temperature, and cooling rate are relevant variables that affect the properties of the final material and, in turn, the supramolecular architecture of the gel network [7,8,9]. The most common features observed in (*R*)-12-HSA gels are the presence of fibers or spherulitic aggregates [7,9]. The former, typically obtained in apolar solvents at slow cooling rates, are associated to gels with better mechanical properties. Spherulites appear in more polar solvents at faster cooling rates. The different mesoscopic aggregates may also correspond to alternative polymorphic forms of the crystalline state. Polymorphism is a widespread phenomenon [10] with remarkable practical implications. In lipids, polymorphism is at the basis of the unpleasant blooming of chocolate due to a structural transition of the triglycerides of cocoa butter [11]. Extensive studies on polymorphic crystal forms of fatty acids highlighted the influence of chain length and crystallization conditions [12,13].

Despite the investigative efforts carried out by various research groups, a complete understanding of factors driving the formation of different aggregates and their macroscopic properties is still lacking. X-ray scattering techniques are commonly exploited due to the partially crystalline character of (*R*)-12-HSA gel networks [1]. Small angle X-ray scattering (SAXS), wide-angle X-ray scattering (WAXS), and X-ray diffraction (XRD) measurements on various specimens afforded the stacking distance of layers of molecules and the interchain distances. It has been reported that the fibers formed in hexane have a hexagonal arrangement of the alkyl chains with an interlamellar distance of approximately 54 Å whereas the spherulites formed in acetonitrile are characterized by a triclinic arrangement with an interdigitation of the lamellar structure of 38−44 Å. In both cases, the interlamellar distance is much larger than the length of the molecule (approximately 26 Å) [14]. However, detailed models of these supramolecular architectures with a description of the intermolecular interactions are still missing. Despite the efforts, a single crystal structure of (*R*)-12-HSA could not be obtained due to its gelation properties at low concentration. On the contrary, the higher concentration required to form organogels of the racemic mixture allowed for crystallization and diffraction analysis [15,16], thus apparently confirming the commonplace that “gelation is crystallization gone wrong” [17]. Recently, we solved the X-ray structure of the positional analogue (R)-9-HSA, which showed less organogelator properties with respect to (*R*)-12-HSA but crystallizes in solvents of variable polarity and hydrogen bonding properties [18]. The crystal structure obtained for this hydroxystearic acid is characterized by a head-to-tail interdigitated arrangement of molecules, with the longest unit cell axis having a value compatible with the length of the molecule. The molecules form cyclic dimers through hydrogen-bonding interactions of the carboxylic acid heads, while the OH groups are engaged in a periodic sequence of interchain hydrogen bonds. The presence of 1D sequences of hydrogen bonds is a stabilizing factor encountered in crystalline structures of secondary alcohols [19]. We also obtained a second polymorphic form by recrystallization of the molten compound through slow cooling. With the use of a simulated annealing protocol, the analysis of the XRPD pattern of this heat-treated crystals yielded a remarkably different structure with a tail-to-tail arrangement of stacks of bent carboxylic dimers. In this case, the longest cell axis is about twice the length of the molecule. Notably, vibrational circular dichroism suggested the presence of bent dimers as the motif responsible for formation of the gel of both enantiomers of 12-HSA in benzene [20].

In this work, we present the results obtained on (*R*)-10-HSA, a positional isomer bearing the hydroxyl group at an intermediate position between 9 and 12, aiming to shed light on the molecular reasons for the different organogelation/crystallization behaviors of (*R*)-9-HSA and (*R*)-12-HSA. X-ray diffraction and IR spectroscopy were used to investigate the ability of (*R*)-10-HSA to induce gelation in organic solvents with different polarities or to form crystals. As the chemical synthesis of the compound is not straight forward, we took advantage of the recently published preparation route for (*R*)-10-HSA that employs a probiotic bacterium, commonly available at as an over-the-counter pharmaceutical preparation able to yield a product with excellent enantiomeric excess through hydration of oleic acid [21]. The industrial interest for (*R*)-10-HSA stems from the possible application of this precursor for the preparation of bio-based polymers [22] and for the synthesis of flavors [23] beside the better-known uses in cosmetics [24]. In this field, (*R*)-10-HSA looks extremely interesting owing to its two-fold action, namely as an active ingredient and texture modifier, thanks to its potential thickening and organogelling abilities. Moreover, endogenous ester derivatives of HSA isomers, including (*R*)-10-HSA, present in mammalian cells have shown antidiabetic and anti-inflammatory properties [25].

## 2. Results

(*R*)-10-HSA affords easily good-quality crystals. They could already be found in the test tubes of the fraction collected from the chromatographic column, after slow evaporation of the solvent, a mixture of petroleum ether and ethyl acetate. The melting point of the crystals is 82.5–84.3 °C, but the measurement carried on after cooling the melt shows a lowering to 78.2 °C, suggesting that crystallization from the melt occurs in a different form, as confirmed by IR spectroscopy and XRPD (see below).

### 2.1. (R)-10-HSA Behavior in Paraffin Oil, Cyclohexane, and Acetonitrile

**Figure 2:**
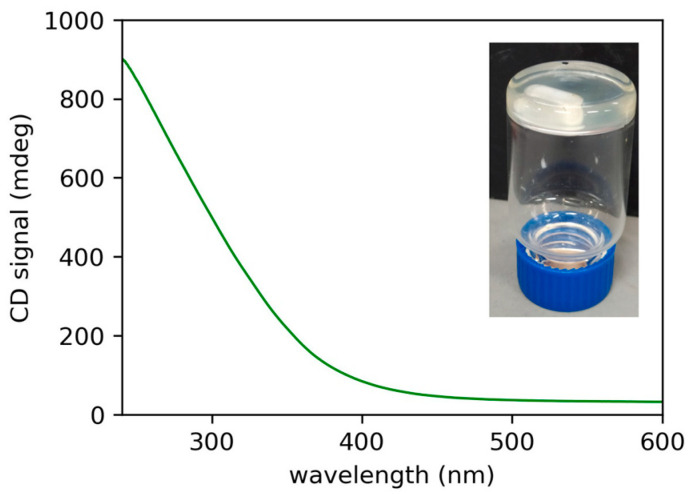
Circular dichroism (CD) spectrum from a layer of the (*R*)-10-HSA 1.8% *w*/*w* gel in paraffin oil. In the inset, the inversion test on the same gel sample.

We tested the organogelator ability using a few solvents where (*R*)-12-HSA is known to gel. We started from paraffin oil, in which (*R*)-9-HSA gives a gel as well, although not stable in time. In detail, the gel was prepared by dissolving (*R*)-10-HSA at a concentration of 1.8% *w*/*w* in paraffin oil at high temperature and by leaving the solution at room temperature for cooling, i.e., following the fast-cooling protocol. The formation of the gel was assessed by the inversion test (Figure 2 inset), and it was examined by circular dichroism (CD). The CD spectrum displays a signal with a positive sign, similar to the gels of both (*R*)-9-HSA and (*R*)-12-HSA in the same solvent. Interestingly, it is more stable in time, at least a few weeks, with respect to the analogous gel of (*R*)-9-HSA, which flows already after one week since preparation [18].

**Figure 3:**
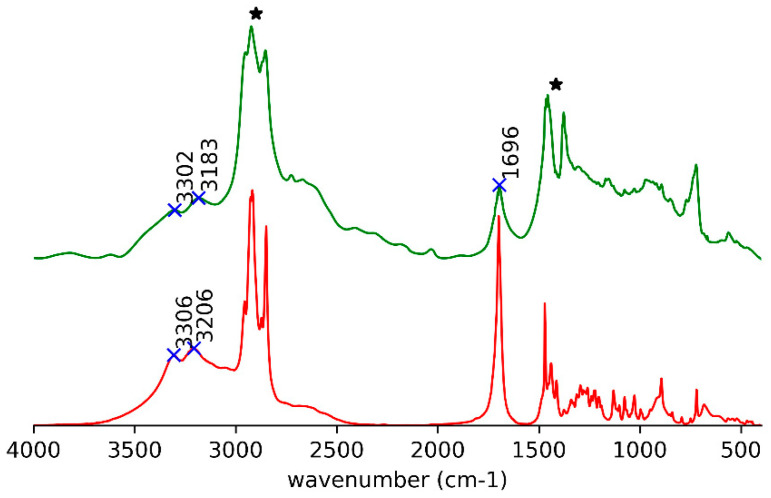
IR spectra for gel in paraffin oil (green trace with the strongest signals, attributed to paraffin oil, marked by asterisks) and heat-treated (*R*)-10-HSA (red trace).

In the IR spectrum, the signals of O–H and C=O stretching modes [26] for (*R*)-10-HSA in the paraffin oil gel occur at the same position and with the same shape as the heat-treated (*R*)-10-HSA (Figure 3).

After the success obtained in paraffin oil, we tested two other solvents, cyclohexane and acetonitrile, following again the procedure described above.

In cyclohexane, which is gelled by (*R*)-12-HSA but not by (*R*)-9-HSA at a concentration of 1% *w*/*w*, we obtained a white, rather fluffy, precipitate (Supplementary Materials Figure S2), so that it occupies half of the volume of the liquid and is stable at least for days.

In acetonitrile, the behavior is somewhat different from (*R*)-9-HSA. Namely, macroscopic white spherical particles appear upon cooling the solution (0.5% *w*/*w*). At the microscopic level, they appear as spherulites, with an apparent dark color because they are compact and do not let light through. They are sparse and coexist with crystals that become more and more abundant, while the spherulites dissolve within about 20 min (Figure S3). On the contrary, spherulites of (*R*)-9-HSA formed in acetonitrile look very light, float in the solution, and quickly grow in volume and number so that they fill all the space, before giving rise to a precipitate of needlelike crystals [18].

**Figure 4:**
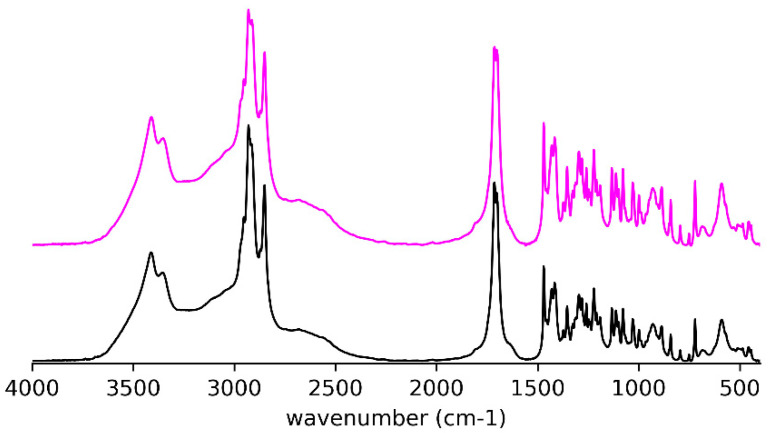
IR spectra for (*R*)-10-HSA from acetonitrile (pink trace) and (*R*)-10-HSA crystals from a mixture of petroleum ether and ethyl acetate (black trace).

The crystals precipitated from the acetonitrile solution have the same IR spectrum (Figure 4) as the crystals used for determination of the single-crystal X-ray structure (see below) and obtained from a mixture of petroleum ether and ethyl acetate.

### 2.2. IR Spectra

**Figure 5:**
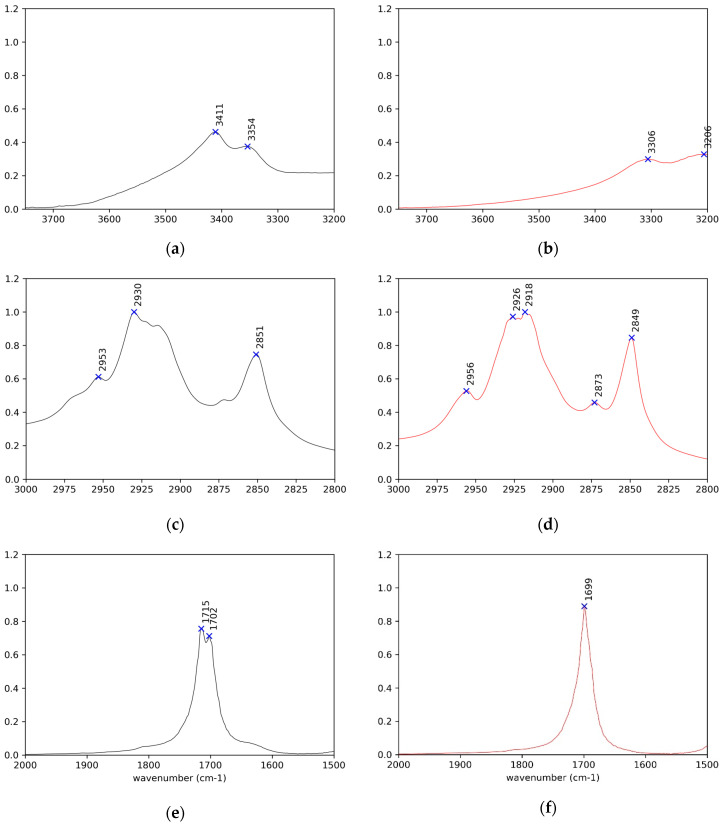
Enlargement of the IR spectra in the regions of OH, CH, and C=O stretching modes respectively (**a**,**c**,**e**) for (*R*)-10-HSA crystals, on the left in black, and (**b**,**d**,**f**) for the heat-treated sample, on the right in red.

**Figure 6:**
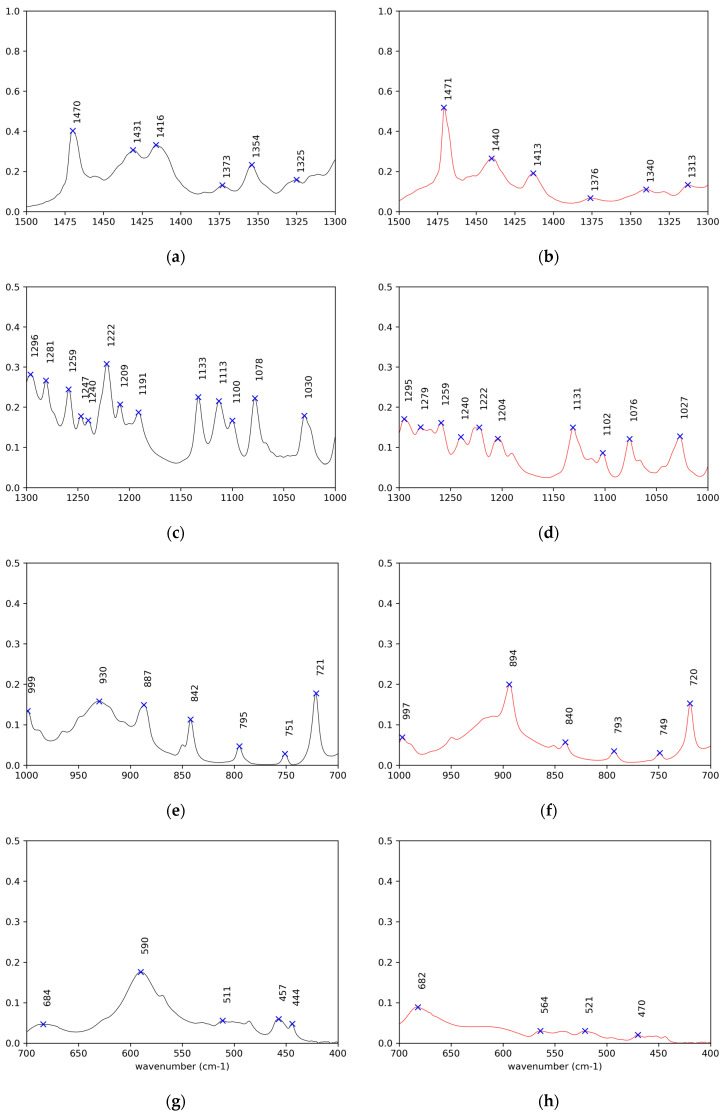
Enlargements of the IR spectra in the fingerprint region (**a**,**c**,**e**,**g**) for (*R*)-10-HSA crystals, on the left in black, and (**b**,**d**,**f**,**h**) for the heat-treated sample, on the right in red.

Detailed comparative examination of the IR spectra of the crystals and solids obtained after thermal treatment of both (*R*)-10-HSA (Figure 3, Figure 4, Figure 5 and Figure 6) and (*R*)-9-HSA (Figures S4 and S5) allowed to single out the peaks more suited to differentiating the various forms. It must be mentioned that, for the IR spectra of the heat-treated sample, a fast cooling of the melt was obtained by keeping at room temperature the plastic test tube containing the crystals previously molten in a water bath.

The assignments were carried out by comparison with the literature data for long-chain fatty acids [13,27,28,29,30] and alcohols [31,32], including data on (*R*)-12-HSA [20].

Overall, the IR spectra of the crystals of (*R*)-10-HSA and (*R*)-9-HSA are very close, as expected considering the strong structural analogies revealed by X-ray diffraction, whereas they are remarkably different with respect to those of the heat-treated samples. A selection of IR bands with relevant assignments is reported in Table 1.

The most revealing features of the IR spectra concern the two sites able to interact through hydrogen bonding, namely the carboxyl group and the chain hydroxyl group.

In the spectra of crystals, both the broad OH stretching band at high wavenumbers, underneath the CH stretching signals (Figure 3 and Figure S4), and the position of the C=O stretching at 1700 cm^−1^ reflect the presence of cyclic carboxylic dimers (Figure 5e and Figure S5e). The splitting of the C=O signal can be attributed to the presence of two molecules in the unit cell and to the vibrational interaction of carboxylic groups of different dimers close in space [13].

The peaks of the coupled C–O stretching and COH in plane bending, of the COH out of plane deformation, and of the O–C=O bending are observed at 1430 (Figure 6a and Figure S5g), 930 (Figure 6e and Figure S5k), and 685 cm^−1^ (Figure 6g and Figure S5m) [28], respectively, for both crystal samples, (*R*)-10-HSA and (*R*)-9-HSA. A signal particularly useful in investigating the conformation around the C(1)–C(2) bond is the O–C=O wagging, which varies from 550 cm^−1^ for a torsional angle of 0° and 180° to 620 cm^−1^ for a torsional angle of 90° [27]. For the crystals, this signal was observed at 590 cm^−1^, which is in an intermediate position, in line with the intermediate torsion angle determined in the crystal structure.

In the heat-treated samples, there is still the broad O–H stretching band of the cyclic carboxylic dimers (Figure 3 and Figure S4). The C=O stretching gives rise to a single signal at 1700 cm^−1^ (Figure 5f and Figure S5f). For the (*R*)-9-HSA sample, a shoulder can be distinguished on the high wavenumber side of the C=O stretching signal, suggesting the presence of sideway dimers in analogy to the arachidic acid, which shows a signal at 1731 cm^−1^ [33]. A further difference with the crystals is the peak of the coupled C–O stretching and COH in plane bending occurring at a higher wavenumber by about 10 cm^−1^, i.e., at 1440 cm^−1^ (Figure 6b and Figure S5h). The COH out of plane deformation is shifted to a lower wavenumber with respect to the crystal, i.e., at 910 cm^−1^ for the heat-treated (*R*)-9-HSA (Figure S5n). The same signal is much broader and therefore difficult to localize for (*R*)-10-HSA. The position of the COH out of plane signal is used as diagnostic feature to differentiate the polymorphs of fatty acids, being sensitive to the conformation of the chain [13].

The most remarkable difference with the spectra of crystals is the disappearance of the 590 cm^−1^ band (Figure 6h), previously assigned to O–C=O wagging, indicating that the conformation at C(1)–C(2) has changed. The presence/absence of this signal can be used as a diagnostic of the polymorphic forms.

Concerning the vibrational modes that involve the chain OH group, the spectrum clearly displays a strong band at high wavenumbers for O–H stretching. For all examined samples, it appears as a double band with a separation of about 60 cm^-1^ (Figure 5a,b and Figure S5a,b). In the case of the crystal, this is due to the participation of the OH group in a long chain of hydrogen bonds. The same might hold for the heat-treated samples, although the presence of two types of hydrogen bonds should not be excluded [34]. The average position, according to the well-known experimental trend with the distance of the hydrogen-bridged oxygen atoms (O–H···O) [35], suggests a hydrogen bond of moderate strength, in agreement with the values found for those distances in the crystals. It is remarkable that the signals shift to lower wavenumber for heat-treated samples, suggesting a strengthening of the side OH hydrogen bonding while keeping the double band shape.

Unfortunately, the other modes involving the chain OH group are superposed with those of the carboxylic acid, preventing their observation. Also, C–OH stretching cannot be unequivocally assigned on the basis of the literature, being the reported range for secondary alcohols 1205–1125 cm^−1^ and envisaged at 1134 cm^−1^ in the case of racemic nonacosan-10-ol [32] but located at 1078 cm^−1^ for (*R*)-12-HSA, with the support of theoretical calculations. In the present samples, two signals at about 1131 cm^−1^ and 1077 cm^−1^ are observed in all spectra (Figure 6c,d and Figure S5i,j) and are almost invariant.

IR spectroscopy can afford information about the conformation of the alkyl chains, starting from the peak of CH stretching of the methylene chain. For a perfect planar, all-trans chain, symmetric stretching is observed at wavenumber values lower than 2922 cm^−1^ and asymmetric stretching is observed at wavenumber values lower than 2852 cm^−1^ [34] (Figure 5c,d and Figure S5c,d). Deviations appear particularly in the case of crystals.

A further information that can be gained from the IR spectra regards the sub-lattice. In the case of orthorhombic perpendicular packing of the alkyl chains, the closer chains interact, causing a split of the CH_2_ scissoring and CH_2_ rocking signals at 1470 cm^−1^ (Figure 6a,b and Figure S5g,h) and 720 cm^−1^ (Figure 6e,f and Figure S5k,l), respectively [28]. This split is not visible either in the spectra of crystals or in those of the heat-treated samples, ruling out the orthorhombic perpendicular sub-cell.

### 2.3. X-ray Single Crystal Structure

**Figure 7:**
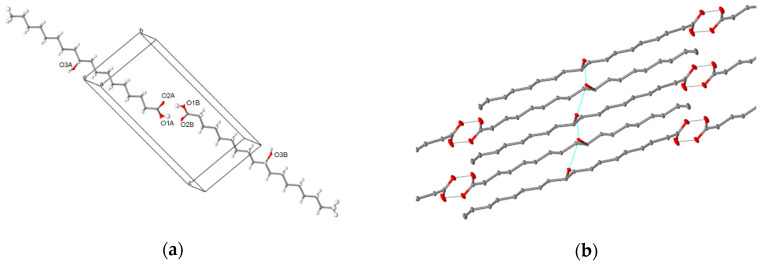
Crystal structure of (*R*)-10-HSA crystallized from a mixture of petroleum ether and ethyl acetate: (**a**) crystallographically independent unit, forming a dimer through hydrogen bonding interactions of the carboxylic moieties and (**b**) hydrogen-bonding interactions involving the hydroxyl group at position 10 of the alkyl chain leading to head-to-tail packing of molecules in the crystal.

Crystals of (*R*)-10-HSA were grown from a mixture of petroleum ether and ethyl acetate, directly after chromatographic separation. Diffraction data from a single crystal were collected at room temperature and at 100 K, with a Mo X-ray tube and synchrotron radiation, respectively. While the first dataset was analyzed only to obtain unit cell parameters (a = 4.974(5) Å, b = 9.159(5) Å, c = 21.22(2) Å, α = 83.89(5)°, β = 88.06(7)°, and γ = 82.69(6)°), the second yielded a structure at a resolution of 0.7 Å (a = 4.835(2) Å, b = 9.150(1) Å, c = 20.725(3) Å, α = 83.097(6)°, β = 89.85(2)°, and γ = 83.097(6)°). Small differences in cell parameters are related to thermal expansion. As expected for an enantiomerically pure sample, crystals of (*R*)-10-HSA belong to the non-centrosymmetric *P1* space group and the refined structure contains two independent molecules, forming a dimer through hydrogen-bond interactions of the carboxylic groups (Figure 7a), with O–O distances of 2.645(1) and 2.661(1) Å. These short values prove the strong interaction between the carboxyl groups. The extended alkyl chains of the two molecules, all in trans conformation, are rotated with respect to each other and their average planes, calculated considering all carbon atoms of each molecule, indicating a 40° rotation (Figure S6). The crystal is formed by the alternate packing of dimers, held together by hydrogen bonds formed by the hydroxyl moieties of molecules oriented in an antiparallel head-to-tail disposition (Figure 7b). The hydrogen bonds involving the hydroxyl groups, having distances of 2.750(1) and 2.752(1) Å for the two independent 10-HSA molecules, respectively, form a chain along the *a* crystallographic direction that interconnects neighboring dimers.

### 2.4. Powder Diffraction Analysis

**Figure 8:**
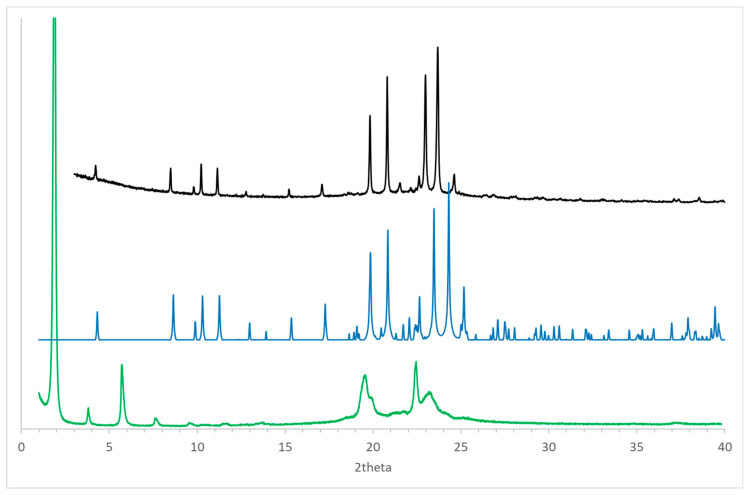
Powder diffraction patterns of (*R*)-10-HSA crystallized from cyclohexane (black line) and after the thermal treatment (green line) at room temperature: the simulated pattern obtained using the single-crystal structure at 100 K has been added for comparison (blue line). Values of 2theta have been calculated considering an incident radiation of 1.55 Å.

Considering the presence of polymorphs observed for positional isomers of (*R*)-10-HSA, such as 12-HSA and 9-HSA [7,8,14,18], the compound was crystallized from a supersaturated cyclohexane solution and analyzed using powder diffraction techniques at room temperature (Figure 8, black line). The comparison of the resulting pattern with the simulated pattern obtained from the single-crystal structure at 100 K (Figure 8, blue line) shows a clear similarity between the two forms. The small differences, particularly visible at 2theta values larger than 20°, can be attributed to the difference in temperature that causes a non-isotropic variation of unit cell parameters.

Thermal treatment of the sample up to the melting temperature and slow cooling led to formation of a different polymorph of (*R*)-10-HSA. In fact, a clear difference in the diffraction pattern is visible as compared to the other crystal forms, with a strong peak at low 2theta values that indicates doubling of the longest unit cell axis of the initial polymorph (Figure 8, green line).

The broader shape of peaks in the diffraction pattern observed for the sample after thermal treatment, compared to that obtained by solvent-assisted crystallization, suggests the formation of smaller and possibly not well-ordered crystals in the former. This effect is particularly visible in peaks as 19.6 and 23.2° of 2theta. However, the sharper peak at 22.4° of 2theta in the pattern of the heat-treated sample may indicate the presence of an additional polymorph, formed during the cooling of the melted sample. Further investigation will be required to assess the effect of the cooling rate on formation of different polymorphs.

## 3. Discussion

### Polymorphs of (R)-10-HSA

The macroscopic behavior of hydroxylated derivatives of stearic acid strongly depends on the position of the derivatization, as proved by the different rheological properties of these compounds. Similarly, the position of the hydroxyl group affects packing in the solid state. Also, as observed in the case of (*R*)-9-HSA [18], different polymorphs are obtained when crystallization is carried out with a solvent with a different polarity and from the melted compound.

In the case of (*R*)-10-HSA, the crystal form obtained from low polarity solvents (petroleum ether and ethyl acetate) shows a head-to-tail arrangement of molecules that seems to be retained when the crystallization is carried out in a more polar solvent such as cyclohexane. A similar behavior was observed also for (*R*)-9-HSA crystallized in the presence of polar or mildly polar solvents. However, a nonpolar solvent such as paraffin yielded a different crystal form for (*R*)-9-HSA.

**Figure 9:**
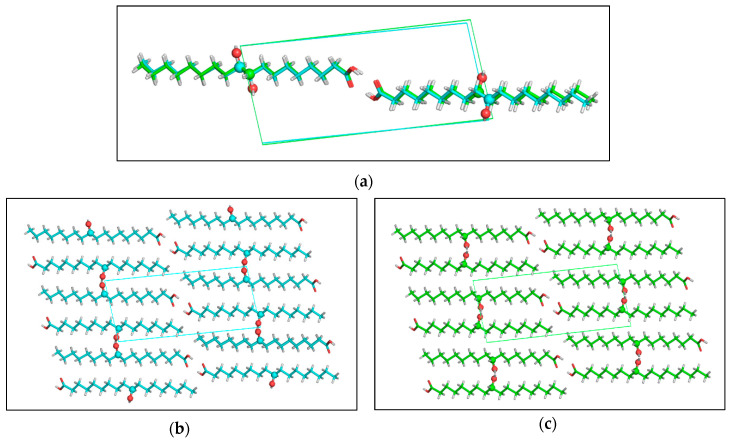
Comparison between the structures of (*R*)-10-HSA and (*R*)-9-HSA crystallized from polar or mildly polar solvents: (**a**) the superimposition of dimers formed by hydrogen-bonding interactions of the carboxylic groups shows that the alkyl chains adopt the same extended conformation for both isomers. (**b**,**c**) Also, the alternate head-to-tail disposition of the molecules in the crystal packing is the same for (**b**) (*R*)-10-HSA as for (**c**) (*R*)-9-HSA. Molecules are represented as sticks, except for the oxygen atoms of the hydroxyl groups and the carbon atoms carrying the derivatization, that are depicted as spheres.

The comparison between single-crystal structures of the two isomers, (*R*)-10-HSA and (*R*)-9-HSA, crystallized from polar and mildly polar solvents, reveals not only an analogous conformation of all-trans elongated alkyl chains (Figure 9a) but also a strikingly similar packing of the molecules in the crystal (Figure 9b,c). In both structures, dimers are held together by strong hydrogen-bonding interactions between the carboxylic heads while hydroxyl moieties of each layer of molecules interact with the same polar groups of the neighboring layer, forming chains of hydrogen bonds extending along the *a* crystallographic direction.

The spatial arrangement of the molecules in these crystal forms is strongly affected by the position of the hydroxyl group, as the head-to-tail pairing of neighboring molecules would be hampered if the lengths of the alkyl chains at opposite sides of the hydroxyl group were significantly different. This is the case, in particular, of the well-known isomer (*R*)-12-HSA, for which a similar crystal form could never be isolated despite the numerous studies focused on this organogelator. While in the case of isomers 9 and 10 the number of carbon atoms at the two opposite sides of the hydroxyl group differs only for one unit, for isomer 12, the molecule is divided in a 11-carbon atom chain on the side of the carboxyl group and a 6-carbon chain at the opposite side. This mismatch hampers the formation of a crystal with a head-to-tail arrangement, since this would result in a less efficient packing of molecules.

Interestingly, for both isomers 9 and 10, a different crystal form is obtained after thermal treatment of the compounds. In particular, for (*R*)-9-HSA, similar patterns were obtained from a paraffin solution and after heat treatment. The latter data could be interpreted to yield a powder diffraction structure, refined with the aid of a simulated annealing protocol. This second polymorph is formed by molecules with extended conformation of the stearic acid derivative observed in the crystal structure from polar solvents but packed in a parallel tail-to-tail arrangement. This arrangement is less stable and probably the result of a kinetically controlled process.

**Figure 10:**
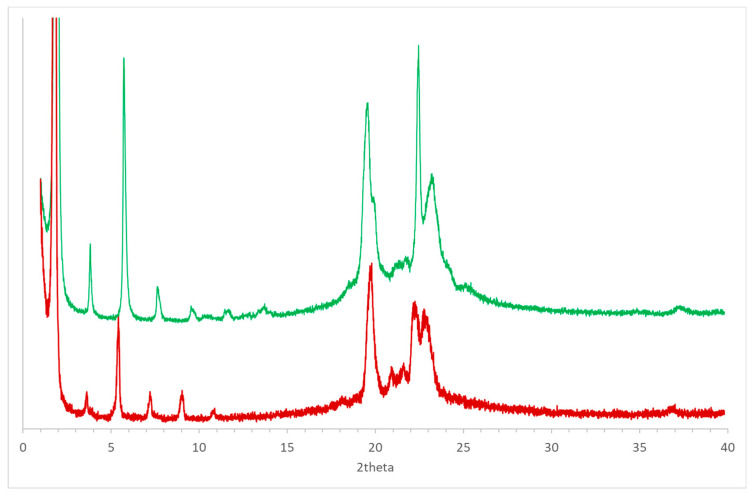
Comparison between the powder diffraction patterns obtained for (*R*)-10-HSA (green line) and (*R*)-9-HSA (red line) [18] after thermal treatment.

A sample of (*R*)-10-HSA underwent a similar treatment, but the diffraction pattern could not be used to obtain a 3D structure. However, the comparison between this pattern and the one obtained after heat treatment of the isomer with the hydroxyl group in position 9 shows a significant similarity, particularly at low values of 2theta (Figure 10). This correspondence suggests that packing of the two isomers in the kinetically controlled polymorph may be analogous, with tail-to-tail arrangements of the molecules. An analogous orientation was suggested for crystalline samples of the (*R*)-12-HSA isomer [9,14]. A tail-to-tail packing of molecules is not significantly affected by the position of the hydroxyl group, since in this case, the parallel orientation of molecules allows the formation of similar interchain hydrogen bonds in all isomers.

## 4. Materials and Methods

### 4.1. Synthesis of (R)-10-Hydroxystearic Acid

(*R*)-10-HSA was prepared on the basis of the literature [21,36] using *Lactobacillus rhamnosus* GG purchased as Dicoflor 60 (AGpharma, Rome, Italy) at a local pharmacy. To 160 mL of sterile de Man-Rogosa-Sharpe (MRS) broth in a 250 Pyrex screw cap bottle, 4 mg of resazurine sodium salt were added; then, the system was fluxed with nitrogen. The content of two sachets of Dicoflor 60 (about 6 × 109 CFU each) suspended in 8 mL of skimmed milk was added to the reaction medium under nitrogen flow. The reaction mixture was incubated for four days at 37 °C and 130 rpm. A solution of oleic acid (480 mg) in ethanol (0.6 mL) and 8 mL of a freshly prepared solution of glucose in water (300 g/L) were added at 3.5 h and 8 h, respectively, since the inoculum is always under nitrogen flow. The reaction mixture was then filtered on celite and acidified to pH 1 with HCl 6N before extracting three times with ethyl acetate. The organic phase was washed with brine and then dried on anhydrous Na_2_SO_4_. After removal of the solvent at reduced pressure, the residue was purified by flash chromatography using petroleum ether/ethyl acetate/acetic acid (9:1:0.05–7:3:0.05) as the eluent.

The enantiomeric excess was determined by ^1^H NMR analysis of the diastereomeric mixture of (R)-O-acetylmandelate esters of the corresponding methyl-10-hydroxysterarates [36,37], resulting at 95% (Figure S1).

### 4.2. Synthesis of (R)-9-Hydroxystearic Acid

(*R*)-9-HSA was obtained as previously reported [18,38].

### 4.3. Gel Preparation

A suspension of (*R*)-10-HSA 1.8 *w*/*w* in paraffin oil was heated in a water bath for 20 min; then, it was left to cool [18].

### 4.4. Circular Dichroism Measurements

The circular dichroism (CD) spectrum was recorded by a JASCO J-710 spectropolarimeter. Owing to the high intensity of the signal of the (*R*)-10-HSA 1.8 *w*/*w* gel in paraffin oil, the spectrum was recorded on a layer obtained by casting 0.9 g of the same hot solution on one of the inner optical faces of a 1-cm optical path, quartz cell.

### 4.5. IR Spectroscopy

The IR spectra were acquired by a Perkin Elmer system 2000R FT-IR spectrometer, accumulating 32 scans with an instrumental resolution of 4 cm^−1^ and a digital resolution of 1 cm^−1^ from KBr pellets. In the case of the gel, it was smeared on a KBr pellet. The spectra are represented as absorbance with the highest peak normalized to unity.

### 4.6. Optical Microscopy

Images were recorded by a Stereozoom Classmag 51 Trino Motic optical microscope equipped with a Moticam 2 digital camera (Motic, Hong Kong, China).

### 4.7. Single-Crystal X-ray Diffraction

Single crystals suitable for an X-ray investigation were obtained by slow evaporation of a petroleum ether/ethyl acetate solution of the (*R*)-10-HSA. Data collection was carried out at the Macromolecular crystallography XRD1 beamline of the Elettra synchrotron (Trieste, Italy), employing the rotating-crystal method with a Dectris Pilatus 2M area detector. Single crystals were dipped in paratone cryoprotectant, mounted on a nylon loop, and flash-frozen under a nitrogen stream at a 100 K. Two datasets of a full 360° crystal rotation in two different crystal orientations were collected from the same crystal. Diffraction data were indexed and integrated using the XDS package [39], while scaling was carried out with XSCALE [40]. The structure was solved using the SHELXT program [41]. The asymmetric unit of the non-centrosymmetric triclinic crystals is composed of two (*R*)-10-HSA molecules. The structure was refined with SHELXL-18/3 [42], operating through the WinGX GIUI [43], by full-matrix least-squares (FMLS) methods on F^2^. Non-hydrogen atoms were refined anisotropically. Hydrogen atoms were added at the calculated positions and refined using the riding model. The absolute chirality of the non-centrosymmetric P1 crystal was determined by the Flack parameter of −0.16(8) using 4616 quotients (I^+^-I^−^)/(I^+^+I^−^). Crystallographic data and refinement details are reported in Table 2.

### 4.8. X-ray Powder Diffraction

To evaluate the possible formation of polymorphic crystalline forms of (*R*)-10-HSA, the sample was crystallized from a supersaturated solution in cyclohexane. The product, not suitable for single-crystal analysis, was measured by X-ray powder diffraction.

The same initial sample was also recrystallized after melting, following slow cooling in a PCR thermocycler: after melting for 12 min at 90 °C, the sample was kept for 30 min at each of the following temperatures 85 °C, 82 °C, 80 °C, 78 °C, 76 °C, 74 °C, 72 °C, 70 °C, and 65 °C and was, finally, cooled down to 20 °C.

Both the powder patterns of (*R*)-10-HSA crystallized from cyclohexane and (*R*)-10-HSA recrystallized after thermal treatment were measured at the MCX beamline [44] of Elettra—Sincrotrone Trieste, (Italy) in transmission mode at room temperature (25 °C) with a monochromatic wavelength of 1.55 Å (8 keV) and 1.033 Å (12 keV) for the sample from cyclohexane and for the heat-treated sample, respectively, and a 1 × 0.3 mm^2^ spot size. The samples were loaded and packed in a 0.5-mm boron capillary, mounted on a standard goniometric head, and spun during data collection. The diffraction patterns were recorded through a scintillator detector in the 2–65° and 1–55° 2theta range for (*R*)-10-HSA crystallized from cyclohexane and (*R*)-10-HSA after heat treatment, respectively.

## Figures and Tables

**Table 1:** IR bands with assignment.

	(*R*)-10-Hsa Crystals	(*R*)-10-Hsa Heat-Treated	(*R*)-9-Hsa Crystals	(*R*)-9-Hsa Heat-Treated
OH stretching	3411, 3354	3306, 3206	3408, 3349	3326, 3234
C=O stretching	1715, 1702	1699	1716, 1698	1702
CH_2_ scissoring	1470	1471	1470	1470
C–O stretching and COH in plane bending	1430	1440	1431	1438
COH out of plane deformation	930	910	929	-
CH_2_ rocking	720	720	722	720
O–C=O bending	685	-	685	-
O–C=O wagging	590	-	592	-

**Table 2:** Crystal data and structure refinement for (*R*)-10-HSA, crystallized from a mixture of petroleum ether and ethyl acetate.

	(*R*)-10-HSA from Petroleum Ether/Ethyl Acetate
Empirical formula	C_18_H_36_O_3_
Formula weight	300.47
Temperature (K)	100(2)
Wavelength (Å)	0.7
Crystal system	Triclinic
Space group	*P* 1
Unit cell dimensions (Å, °)	*a* = 4.835(2), a = 83.097(6)
	*b* = 9.150(1), *β* = 89.85(2)
	*c* = 20.725(3), g = 83.097(6)
Volume (Å^3^)	901.1(4)
Z	2
*ρ*_calc_ (g/cm^3^)	1.107
*μ* (mm^−1^)	0.07
F(000)	336
Reflections collected	31,174
Independent Reflections	9878
restraints/parameters	3/386
GooF	1.04
Final R indices (I > 2σ(I))	R_1_ = 0.0278, wR_2_ = 0.0801
R indices (all data)	R_1_ = 0.0278, wR_2_ = 0.0801
CCDC code	2025781

## Supplementary Materials

Supplementary materials can be found at https://www.mdpi.com/1422-0067/21/21/8124/s1.
