# Supplementary material for: (R)-10-Hydroxystearic Acid: Crystals vs. Organogel
Source: Int J Mol Sci. 2020 Oct 30;21(21):8124. doi: 10.3390/ijms21218124 (PMC7662707; doi:10.3390/ijms21218124)
Supplement: Supplementary file 1 [file ijms-21-08124-s001.pdf]

International Journal of
Molecular Sciences

MDPI

(*R*)-10-Hydroxystearic Acid: Crystals vs. Organogel

Fioretta Asaro, Carla Boga, Rita De Zorzi, Silvano Geremia, Lara Gigli, Patrizia Nitti and Sabrina Semeraro

Supplementary Material

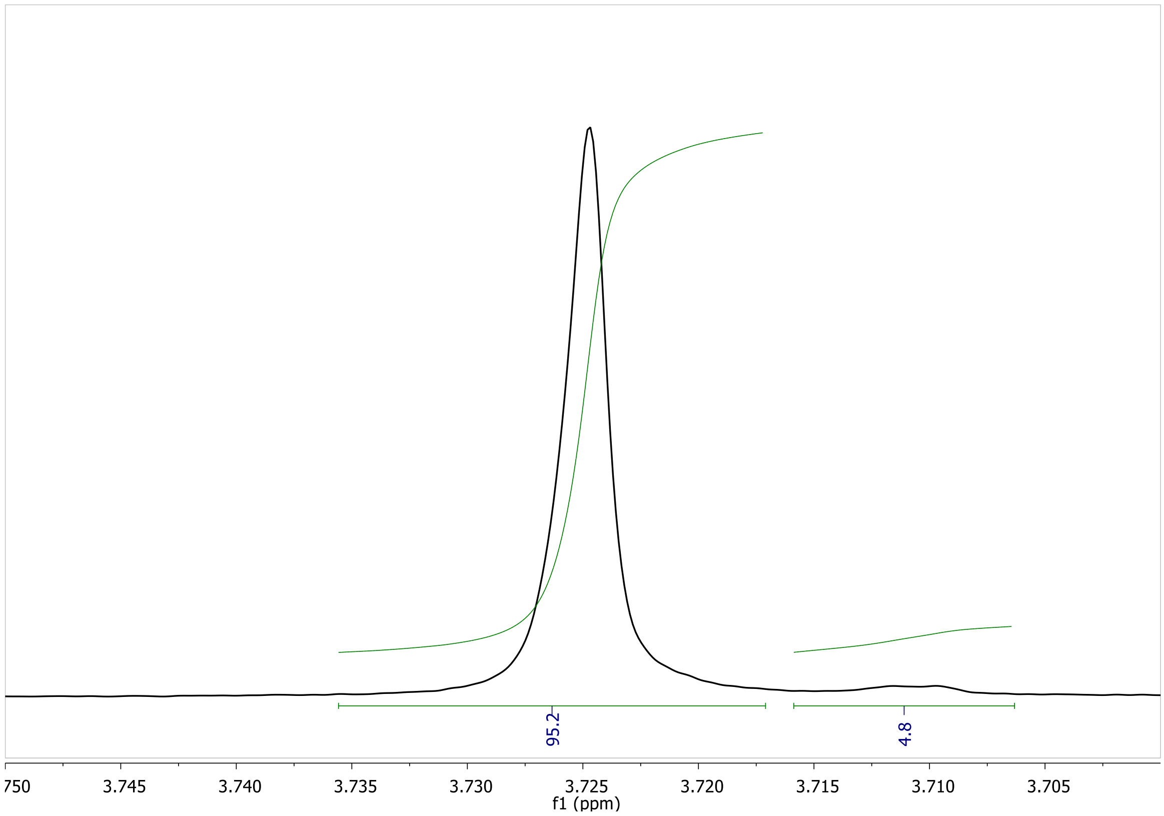

Figure S1. ^{1}H NMR (500 MHz) analysis of the diastereomeric mixture of the (*R*)-O-acetylmandelate esters obtained from the methyl esters of (*R*)-10-hydroxystearic acid ((*R*)-10-HSA). The figure shows the signal of the methoxy group [1,2].

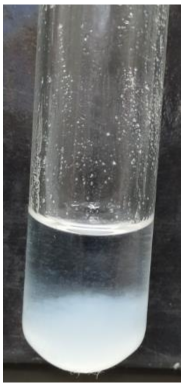

Figure S2. (*R*)-10-HSA (1% *w*/*w*) in cyclohexane.

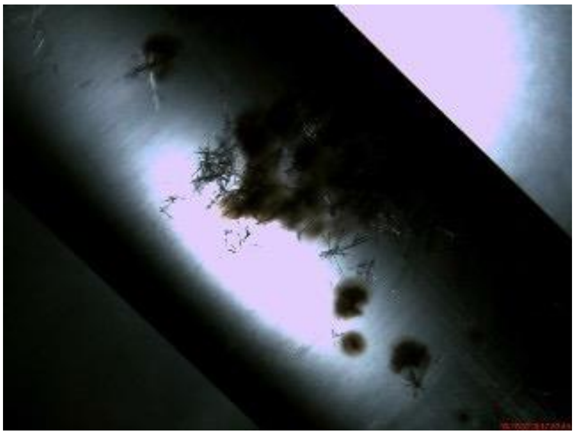

(**a**)

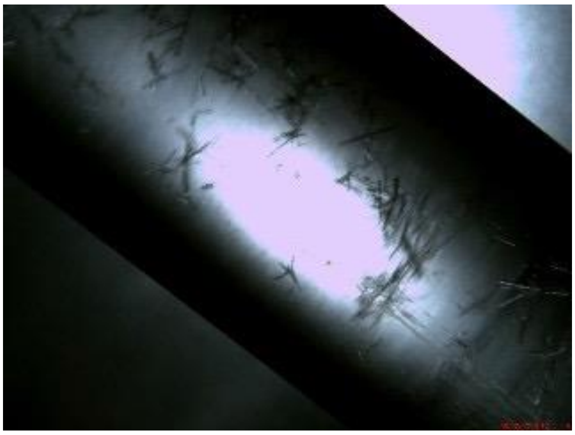

(**b**)

Figure S3. Images obtained with an optical microscope showing the evolution of (*R*)-10-HSA 0.5% w/w in cyclohexane in a 10 mm test tube: (a) spherulites, (b) crystals. The images were taken 15 minutes apart.

(R)-9-Hydroxystearic acid IR spectra

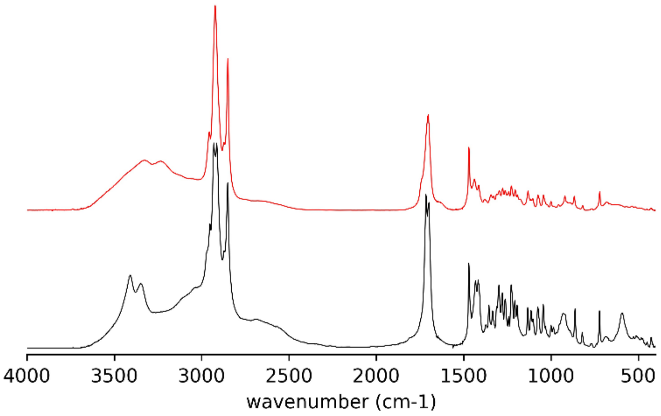

Figure S4. IR spectra for (*R*)-9-HSA crystals (black trace) and the same heat-treated sample (red trace).

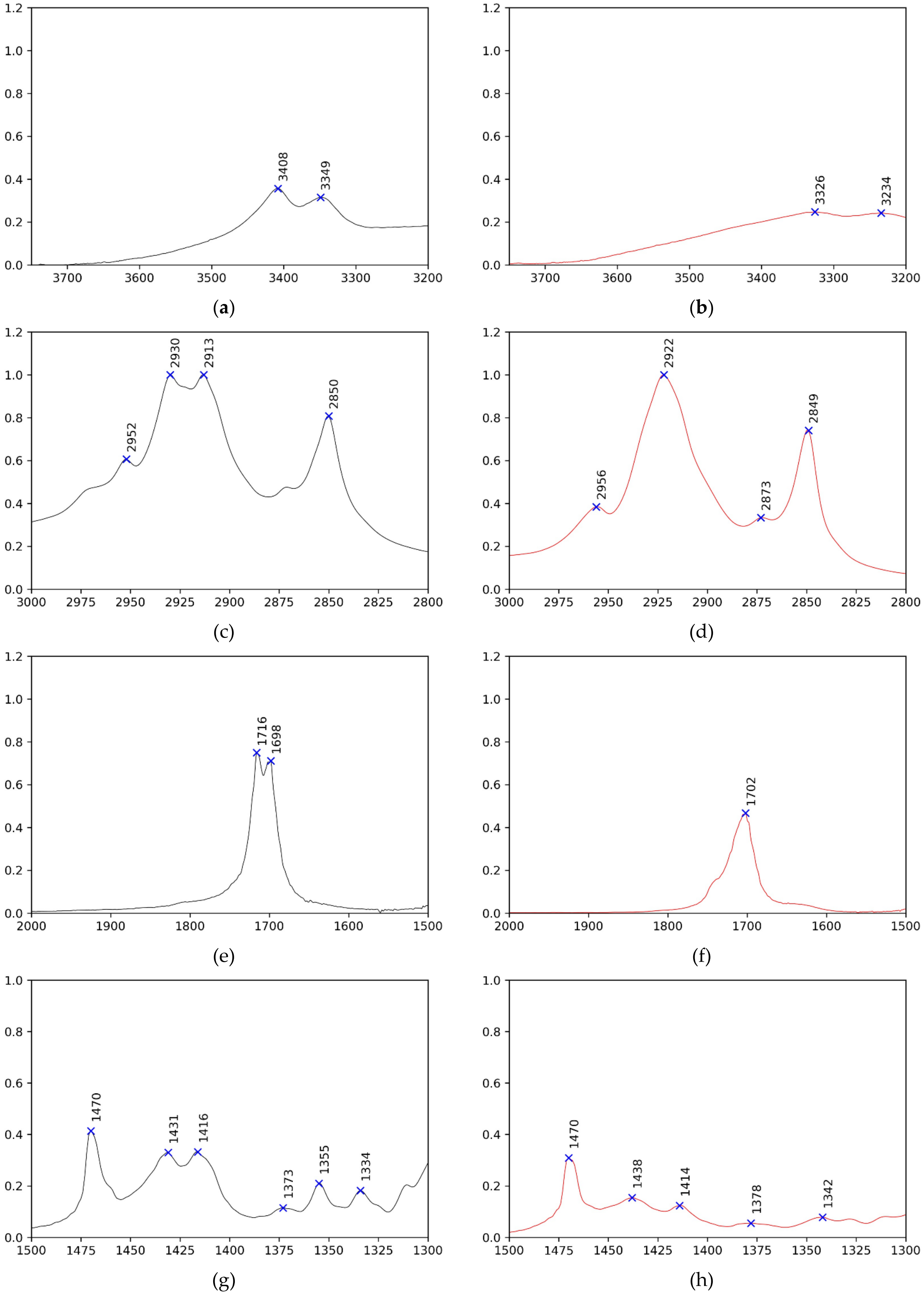
3408
3349
(**a**)
3326
3234
(**b**)
2952
2930
2913
2850
(c)
2956
2922
2873
2849
(d)
1716
1698
(e)
1702
(f)
1470
1431
1416
1373
1355
1334
(g)
1470
1438
1414
1378
1342
(h)

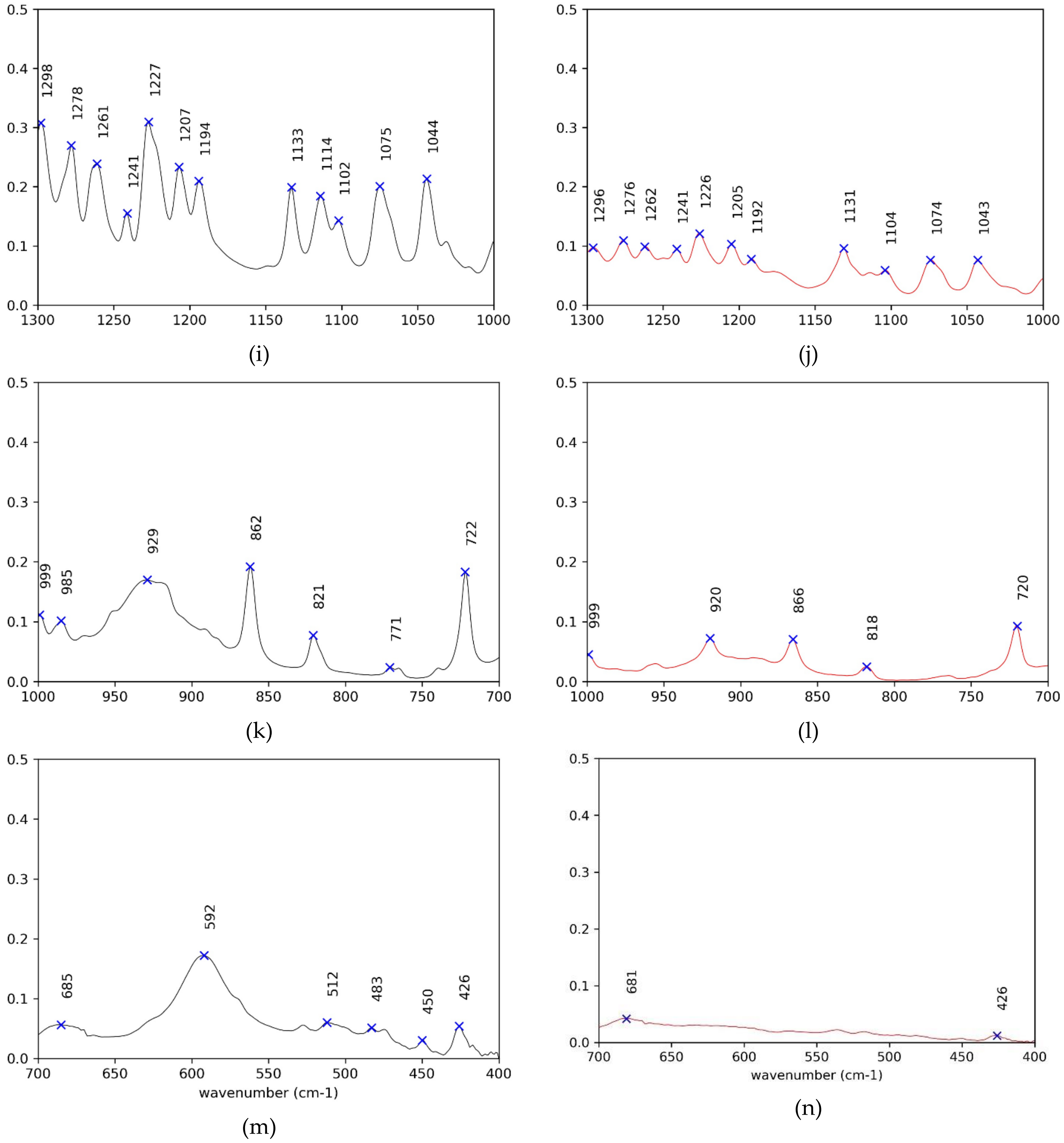

Figure S5. Enlargement of the IR spectra for the (*R*)-9-HSA crystals (in black on the left) and the same heat-treated sample (red on the right).

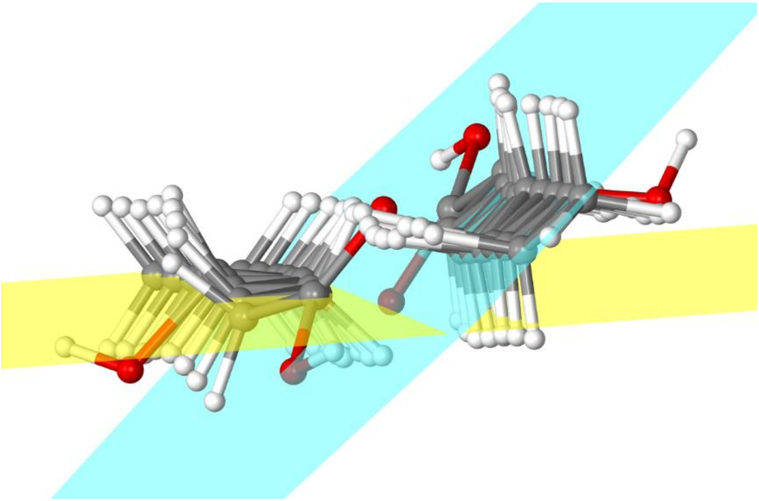

Figure S6. Crystal structure of (*R*)-10-HSA crystallized from petroleum ether and ethyl acetate. The average planes of the two alkyl chains are rotated with respect to each other, leading to an out-of-plane conformation of the dimer.

References

1. Stefano Serra; Davide De Simeis Use of Lactobacillus rhamnosus (ATCC 53103) as Whole-Cell Biocatalyst for the Regio- and Stereoselective Hydration of Oleic, Linoleic, and Linolenic Acid. *Catalysts* **2018**, *8*, 109, doi:10.3390/catal8030109.
2. Yang, W.; Dostal, L.; Rosazza, J.P.N. Stereospecificity of Microbial Hydrations of Oleic Acid to 10-Hydroxystearic Acid. *Appl. Environ. Microbiol.* **1993**, *59*, 281–284, doi:10.1128/AEM.59.1.281-284.1993.
